# Excess glutamine in embryo culture media alters mitochondrial and autophagy-related dynamics associated with impaired postnatal development

**DOI:** 10.3389/fcell.2026.1920196

**Published:** 2026-09-17

**Authors:** Norermi Firzana Alfian, Yui Maruhashi, Masahiro Horizoe, Wasim Bari, Shiori Ishiyama, Kazuki Mochizuki, Satoshi Kishigami

**Affiliations:** 1 Department of Integrated Applied Life Science, Integrated Graduate School of Medicine, Engineering, and Agricultural Sciences, University of Yamanashi, Kofu, Yamanashi, Japan; 2 Faculty of Life and Environmental Sciences, University of Yamanashi, Kofu, Yamanashi, Japan; 3 Graduate School of Life and Environmental Sciences, Integrated Graduate School of Medicine, Engineering and Agricultural Science, University of Yamanashi, Kofu, Yamanashi, Japan; 4 Center for Advanced Assisted Reproductive Technologies, University of Yamanashi, Kofu, Yamanashi, Japan

**Keywords:** autophagy, DOHaD, embryo culture, embryogenesis, glutamine, mitochondria

## Abstract

The preimplantation period is a critical nutrient-sensing window influencing metabolic disease risk, as established by the developmental origins of health and disease theory (DOHaD). In this study, we examined the postnatal consequences of increased glutamine concentration, an important energy source, by altering embryonic metabolic development. *In vivo*-fertilised ICR mouse embryos were cultured in modified Chatot-Ziomek-Bavister medium with 1 mM L-glutamine (Gln) and bovine serum albumin (BSA) as the control and compared with elevated Gln media (2 mM) with bovine serum albumin (+Gln) or without BSA (+Gln-BSA). Early embryonic developmental analysis showed that both +Gln and +Gln-BSA presented reduced blastocyst cell number, primarily in trophectoderm cells, associated with increased apoptosis. Embryos showed altered metabolic homeostasis, including reduced mitochondrial membrane potential and mitochondrial morphology, altered autophagy-related activity (indicated by discrepancies between the DAPGreen assay and LC3-positive autophagosomes), and reduced heterochromatin methylation signals (H3K27me3). Offspring derived from these embryos subsequently exhibited irregular body weight trajectories, reduced survival, and impaired glucose tolerance. These findings indicate that *in vitro* exposure to elevated glutamine during early development is associated with altered mitochondrial dynamics and autophagy-related responses, interrupting intracellular mechanisms with lifelong metabolic consequences.

## 1 Introduction

Early embryonic development is an extremely sensitive period during which cells actively sense and respond to nutrient availability (Velazquez et al., 2023). This correlation between nutrition and preimplantation embryo development has been confirmed through recent reports broadly based on the Developmental Origins of Health and Disease (DOHaD) framework, which suggests that nutritional exposure during gestation triggers an embryo reprogramming mechanism that could permanently alter bodily functions and metabolic systems (Peral-Sanchez et al., 2021). The nutritional exposure causes a conditional programming that imprints cellular memory, in the form of epigenetic memory and metabolic memory, that eventually manifests into long-term diseases such as obesity and type 2 diabetes in offspring (Alfian et al., 2026).

The *in vitro* culture systems used in assisted reproductive technologies (ART) provide controlled conditions that aim to support *ex utero* development (Brinster, 1963). Recently, we reported that when embryos were cultured *in vitro* from the 2-cell stage to the morula stage using α-minimal essential media (αMEM), rather than the commonly used standard media such as Potassium Simplex Optimisation Medium (KSOM) or Chatot-Ziomek-Bavister (CZB) media, offspring derived from embryo transfer exhibited type 2 diabetes-like phenotypes (MEM mouse model) (Ishiyama et al., 2021a; Ishiyama et al., 2021b). These findings indicate that the culture conditions themselves, particularly medium composition, can influence postnatal health outcomes by altering developmental trajectories, and further suggest that components of αMEM may contribute to the programming of diabetes susceptibility. Notably, αMEM contains various components, including higher levels of glutamine compared to standard media. However, ɑMEM lacks bovine serum albumin (BSA), a component commonly included in culture systems that provides undefined yet supportive factors for embryonic development with postnatal influences (Bavister, 1995; Jharna et al., 2025). Consequently, it remains unclear whether the observed metabolic phenotype arises due to increased glutamine availability or other compositional differences in the medium. This limitation should therefore be explicitly addressed, as it is essential to determine whether a single factor or a combination of factors is causally responsible for the metabolic alterations observed.

Glutamine is consumed at high rates and supports tricarboxylic acid (TCA) (Devreker et al., 1998), nucleotide biosynthesis, redox homeostasis, and amino acid exchange (Brinster, 1971). Previous work has observed the importance of glutamine in embryo development with emphasis on the developmental perturbations (He et al., 2007). It is a key nutrient in many culture media used in commercial *in vitro* media in ART (Summers et al., 2005), and it holds a substitutionary role as a preferred energy source reliant on conditions (Chatot et al., 1989). It has been discussed that prolonged high glutamine consumption results in cellular alterations and substantial deteriorating risks (Holecek, 2013; Villar and Durán, 2017). The reasoning for these alterations is due to the toxicity of ammonia accumulation from glutamine degradation in the culture media, as suggested by previous studies (Kleijkers et al., 2016; Bouillon et al., 2016). The commonly used concentration of L-glutamine in embryo culture media is 1 mM in consideration of the possible ammonia accumulation after long-term storage (Lane et al., 2001) and is consistent with the physiological levels reported in pregnant mice (Eckert et al., 2012). Glutamine-produced ammonia or ammonium itself causes detrimental effects on embryonic development by inducing apoptosis of blastomeres, reducing cell numbers and foetal growth (Sinawat et al., 2003; Lane and Gardner, 1994). The increased consumption of glutamine is associated with declining embryo quality, and the glutamine demands shift depending on embryonic developmental stage (Zuo et al., 2020; Chen et al., 2018; Chatot et al., 1990). A study using cancer cells after exposure to elevated glutamine levels observed a shift in energy source dependency defined as ‘glutamine addiction’ (Bodineau et al., 2022; Bodineau et al., 2021). This possible correlation between glutamine metabolism and cellular homeostasis during the preimplantation period merits careful consideration. Accordingly, investigating the influence of glutamine availability during preimplantation development is essential to clarifying its role in the association with long-term diseases through embryonic metabolic programming.

In this study, we tested the hypothesis that elevated L-glutamine concentrations during the preimplantation window induce permanent metabolic programming, manifesting as irregular body growth and impaired glucose tolerance in adulthood. By applying a standard *in vitro* embryo culture system and embryo transfer technique, we can provide indications of elevated glutamine concentrations in embryo culture and determine whether increased glutamine availability on top of *in vitro* culture conditions during this critical developmental window contributes to the development of metabolic disorders in adulthood. We compared the control against glutamine-supplemented groups with (+Gln) or without BSA (+Gln-BSA). We assessed early developmental conditions through cell number, lineage, and apoptosis. Furthermore, we investigated the mechanistic links during early embryo metabolism, by examining the mitochondria, oxidative stress, autophagy and also linking to glucose intolerance-related epigenetic modifications in heterochromatin methylation H3K27me3. To further observe the implications towards the subsequent risk of developing chronic health conditions, offspring derived from culture conditions were monitored for postnatal growth and glucose homeostasis.

## 2 Materials and methods

### 2.1 Experimental design

To analyse the long-term effects of glutamine on embryo development, embryos were cultured continuously in one of three media conditions 1): control modified CZB medium (mCZB) (Chatot et al., 1990), 2) mCZB supplemented with 2 mM glutamine (+Gln), or 3) mCZB supplemented with 2 mM glutamine in the absence of bovine serum albumin (+Gln-BSA) as shown in Supplementary Table S1. The +Gln-BSA group was included to determine potential influences of the interrelationship between BSA-derived factors and L-glutamine.

### 2.2 Animals

All experimental procedures were conducted following the ethical guidelines of the Guide for the Care and Use of Laboratory Animals and approved by the Institutional Committee of Laboratory Animal Experimentation of University of Yamanashi (protocol number A6-19). All mice were of ICR strain and obtained from the Shizuoka Laboratory Animal Centre (SLC) Inc (Hamamatsu, Japan). Mice were kept under Specific Pathogen-Free (SPF) conditions, with controlled temperature (25 °C), relative humidity (50%), and photoperiod (14L-10D). They were fed *ad libitum* with a standard pelleted diet (MF, Oriental Yeast Co., Ltd., Tokyo, Japan) and distilled water.

### 2.3 Embryo retrieval and *in vitro* culture

Female mice (8–10 weeks of age) were super-ovulated by an injection of 7.5 IU pregnant mare serum gonadotropin (PMSG; ASKA Pharmaceutical Co. Ltd., Tokyo, Japan) followed 48 h later by 7.5 IU human chorionic gonadotropin (hCG; ASKA Pharmaceutical Co. Ltd., Tokyo, Japan). After the second injection, mice were left overnight with male mice and examined for vaginal plugs the following morning. After 5–6 h, mice were euthanised by cervical dislocation and zygotes were collected at the 1-cell or 2-cell stage using Hepes-mCZB medium (Sigma-Aldrich Chemical Co., St. Louis, MO, United States). Droplets of media were placed under lightweight paraffin oil for at least 1 h to equilibrate preliminarily before the experiment. Oocytes were washed three times before *in vitro* culture into their respective treatment groups. To mitigate ammonia accumulation from long-term storage, each culture medium was prepared fresh monthly (Zhao et al., 2016). Embryos were incubated in an atmosphere of 5% CO_2_ in air at 37 °C.

### 2.4 Assessment of preimplantation development

Immunofluorescence was carried out as described previously (Alfian et al., 2025). In brief, embryos were fixed in 4% (w/v) paraformaldehyde (PFA) and permeabilised with 0.1% Triton X-100 and 1% BSA in PBS. Then, embryos were incubated overnight at 4 °C in primary antibodies. Primary antibodies used were anti-CDX2 (1:500, BioGenex, MU392-UC) and anti-Nanog (1:500, Abcam, ab80892). After washing, embryos were incubated with secondary antibodies, Alexa Fluor 488-conjugated anti-rabbit IgG (1:500) and Alexa Fluor 568-conjugated goat anti-mouse (1:500), for at least 3 h at 4 °C. Embryos were mounted on glass slides in Vecta-shield (Vector Laboratories Inc., Burlingame, CA) supplemented with 1 μg/mL 4′,6-diamidino-2-phenylindole (DAPI). The fluorescence signals were observed using a fluorescence microscope (BZX-810; Keyence, Osaka, Japan) for cell number analysis. Cell numbers were manually counted with the ImageJ Fiji software.

### 2.5 Assessment of apoptosis

Embryos were fixed with 4% PFA and permeabilised. Apoptosis in embryos was analysed with the MEBSTAIN Apoptosis TUNEL Kit Direct (MBL, Nagoya, Japan) according to the manufacturer’s instructions. Embryos were counterstained with DAPI for nuclear staining. The fluorescence of the fragmented DNA ends was detected by the confocal laser-scanning microscope (Olympus FV1200; Olympus, Tokyo, Japan), and the apoptotic index was calculated for each embryo as follows (number of TUNEL-positive/Total cell number (TCN)) x 100.

### 2.6 Analysis of mitochondrial function and oxidative stress

#### 2.6.1 Mitochondrial membrane potential

For observation of the mitochondrial membrane potential, morula-stage embryos were stained. Embryos were washed three times in mCZB media and incubated for 30 min in the same medium containing 200 nM Mitotracker™ Red (Invitrogen, Molecular Probes, Oregon, United States) mitochondrial-specific fluorescent dye (Poot, M., et al., 1996) diluted in mCZB media at 37 °C under 5% CO_2_ in air. Embryos were then washed and incubated for over 10 min in mCZB medium containing 1 μg/mL Hoechst 33342 (Dojindo, Kumamoto, Japan). The embryos were transferred to Hepes-CZB drops on a glass bottom dish (35 mm dish; MatTek Corp., MA, United States) for observation. Imaging was conducted by using a confocal fluorescence microscope (Olympus FV1200; Olympus, Tokyo, Japan).

#### 2.6.2 ROS assay

For observation of the reactive oxygen species, embryos were washed three times in mCZB media and incubated for 30 min in the same medium containing 5 µM CellROX™ Green Reagent (Invitrogen, Molecular Probes, Oregon, United States) diluted in mCZB media to make 5 µM medium at 37 °C under 5% CO_2_ in air. Embryos were then washed and incubated for over 10 min in mCZB medium containing 1 μg/mL Hoechst 33342 (Dojindo, Kumamoto, Japan). The embryos were transferred to Hepes-CZB drops on a glass bottom dish (35 mm dish; MatTek Corp., MA, United States) for observation. Imaging was conducted by using a fluorescence microscope (BZX-810; Keyence, Osaka, Japan).

#### 2.6.3 Quantification of mitochondrial volume and morphology

Confocal microscopy (Olympus FV1200; Olympus, Tokyo, Japan) was used to capture images of embryo mitochondria stained using MitoTracker ™ Red (Invitrogen, Molecular Probes, Oregon, United States). The volume of mitochondria was quantified using the Fiji Plugin, ‘Mitochondria Analyzer’ (Chaudhry et al., 2020). Images were converted to 8-bit and three-dimensional (3D) analysis was performed using the settings: max slope: 1.40, gamma: 0.90, block size: 1.25 μm and C-value: 5. ‘Despeckle’, ‘Remove Outliers’ and ‘Fill 3D Holes’ were set to be performed post-processing. ‘Total mitochondrial volume’, ‘mitochondrial count’ and ‘sphericity’ were noted.

#### 2.6.4 Mitochondria-ROS colocalization

For observation of the oxidative stress, embryos were washed three times in mCZB media and incubated for 30 min in the same medium containing 200 nM Mitotracker™ Red (Invitrogen, Molecular Probes, Oregon, United States) mitochondrial-specific fluorescent dye (Poot, M., et al., 1996) and 5 µM CellROX™ Green Reagent (Invitrogen, Molecular Probes, Oregon, United States) diluted in mCZB media to make 5 µM medium at 37 °C under 5% CO_2_ in air. Embryos were then washed and incubated for over 10 min in mCZB medium containing 1 μg/mL Hoechst 33342 (Dojindo, Kumamoto, Japan). The embryos were transferred to Hepes-CZB drops on a glass bottom dish (35 mm dish; MatTek Corp., MA, United States) for observation. Imaging was conducted by using a fluorescence microscope (BZX-810; Keyence, Osaka, Japan).

Colocalization analysis was performed using the JACoP plugin on ImageJ Fiji software (NIH, United States) (BOLTE and CORDELIÈRES, 2006). For each channel, the Cortes threshold was set and used for the data set. The degree of colocalization was reported as a Pearson’s correlation coefficient, quantifying the overlap degree between fluorescence signals. Colocalization of mitochondria onto ROS represents oxidative stress.

### 2.7 Analysis of autophagy-associated process

#### 2.7.1 DAPGreen staining

DAPGreen-Autophagy Detection (DAPGreen) was performed on control and glutamine-treated morula-stage embryos. Autophagosomes and autolysosomes were stained by incubating the embryos with 0.5 µM DAPGreen (Dojindo, Japan) and 200 nM Mitotracker™ Red (Invitrogen, Molecular Probes, Oregon, United States) diluted in mCZB media for 30 min. Embryos were then washed and incubated for over 10 min in mCZB medium containing 1 μg/mL Hoescht33342 (Dojindo, Kumamoto, Japan) at 37 °C under 5% CO_2_ in air. The embryos were transferred to Hepes-CZB drops on a glass bottom dish for observation. Confocal microscopy (Olympus FV1200; Olympus, Tokyo, Japan) was used to capture images of each embryo along the Z-axis in 10 sections.

#### 2.7.2 LC3 staining

Embryos were fixed in 4% (w/v) paraformaldehyde (PFA) and permeabilised with 0.1% Triton X-100 and 1% BSA in PBS. Then, incubated overnight at 4 °C in the primary antibody, anti-LC3 (1:1000, MBL, PM036). After washing, embryos were incubated with the secondary antibody, Alexa Fluor 488-conjugated anti-rabbit IgG (1:500). Confocal digital images of fluorescence signals were captured using a confocal laser-scanning microscope (Olympus FV1200; Olympus, Tokyo, Japan) with consistent laser settings and exposure time for epigenetic observations.

#### 2.7.3 Evaluation of LC3 autophagosomes morphology

Confocal images of LC3-stained embryos were used to analyse LC3 puncta. Analysis of puncta was performed as previously reported (Tatebe et al., 2025). In brief, image stacks were background-subtracted with a rolling ball radius of one pixel and a Gaussian blur filter with a radius of two pixels. Threshold was applied to each section, and 3D object counting was performed with a size limit filter of 5–10,000 pixels. Number of puncta per embryo and the average puncta volume (μm^3^) were quantified and analysed.

#### 2.7.4 Evaluation of mitochondria and autophagosome-autolysosome colocalization representing mitophagy-related process

Colocalization analysis was performed using the JACoP plugin. For each channel, the Costes threshold was set and used for the data set. The degree of colocalization was reported as a Pearson’s correlation coefficient, quantifying the overlap degree between fluorescence signals. Colocalization of mitochondria onto autophagosomes and autolysosomes was analysed to represent mitophagy activity.

### 2.8 Heterochromatin methylation signal analysis

Immunofluorescence was carried out where embryos were fixed in 4% (w/v) paraformaldehyde (PFA) and permeabilised with 0.1% Triton X-100% and 1% BSA in PBS. Then, embryos were incubated overnight at 4 °C in primary antibody. For the primary antibody, we used anti-tri-methyl Lys27 (H3K27me3, 1:500, Genetex inc., GTX121184). After washing, embryos were incubated with secondary antibody, Alexa Fluor 488-conjugated anti-rabbit IgG (1:500) for at least 3 h at 4 °C. Embryos were mounted on glass slides in Vecta-shield (Vector Laboratories Inc., Burlingame, CA) supplemented with 1 μg/mL 4′,6-diamidino-2-phenylindole (DAPI). Confocal digital images of fluorescence signals were captured using a confocal laser-scanning microscope (Olympus FV1200; Olympus, Tokyo, Japan) with consistent laser settings and exposure time for signal observations.

### 2.9 Embryo transfer and postnatal follow-up

Surrogate pseudo-pregnant females that were used as recipients of the embryos were mated with vasectomised males, whose sterility was previously proven. Morula-stage embryos derived from *in vitro* culture media were transferred into oviducts at 0.5 days post-coitus (dpc) after mating. Between 8 and 10 embryos were transferred into each oviduct during each embryo transfer with the number of recipient females used for each experimental group summarized in Supplementary Table S2. Following birth, offspring were reared by their foster dam under normal litter conditions. Offspring produced via natural birth (E19.5) were weighed and monitored weekly until Week 16, the completion of the experiment.

### 2.10 Oral glucose tolerance test (OGTT)

Earlier studies using the MEM mouse model identified metabolic alterations predominantly in male offspring (Ishiyama et al., 2021c). Therefore, the present study focused on males to determine whether there is a similar susceptibility as found in the MEM mouse model. After birth, the offspring were fed a standard diet until 8 weeks of age, after which male mice were fed a high-fat diet (F2WTD, Oriental Yeast Co., Ltd., Tokyo, Japan). The exact contents of the high-fat diet are listed in Supplementary Table S3. Female mice were given a commercial diet throughout the experiment. Mice at 8, 12, and 16 weeks of age were exposed to an oral glucose tolerance test (OGTT). The total fasting period was 6 h before OGTT, while water was still provided *ad libitum*. The first measurement of fasting glucose levels was taken, and then 0.1 mL of 20% glucose was administered per 10 g body weight using an oral gavage. Blood glucose levels were measured at 15, 30, 60, and 120 min intervals after administration from blood samples collected from the tail tip. At 16 weeks, mice were euthanised by cervical dislocation. From the OGTT, a glucose tolerance graph was plotted, and the incremental area under the curve (iAUC) was calculated.

### 2.11 Statistical analysis

Each experimental analysis was performed with three to five independent replicates. Statistical analyses were conducted using GraphPad Prism 10 Version 10.3.1 (2024). Fluorescence data were analysed using ImageJ Fiji Software, and for each embryo, the ROI was defined by outlining the cytoplasmic area based on positive fluorescence signals. For fluorescence intensity calculation, the relative fluorescence unit (RFU) was calculated by subtracting the average background and normalised with the control group. All data are expressed as the mean values ± standard error of the mean (SEM) unless otherwise indicated. All data were analysed using a one-way analysis of variance (ANOVA) followed by Tukey-Kramer’s HSD multiple comparison test unless specifically mentioned. All data presented have been verified for normality and homogeneity of variance. The survival curve was produced by a Kaplan-Meier plot. Preliminary analysis confirmed that litter size did not significantly contribute to overall variance in postnatal body weight data and growth z-score (p > 0.05), therefore it was omitted from the final model to prevent over-parameterization. To identify specific group differences, pairwise differences between groups using Tukey’s *post hoc* multiple comparisons test was performed following the mixed-effect analysis from GraphPad Prism. Significance was taken as p-values with less than 0.05.

## 3 Results

### 3.1 Increased glutamine supplementation alters mouse blastocyst trophectoderm cell specification

To investigate the effects of increased glutamine supplementation in culture media on embryo development, we cultured *in vivo*-fertilized mouse embryos continuously for either 72 or 96 h or transferred back to control medium at the morula stage after 48 h of glutamine exposure (Figure 1A). After the *in vitro* embryo culture, we observed the blastocyst formation and hatching rate in each experimental group and most of the embryos were able to reach the blastocyst stage with no apparent changes in hatching rate (Table 1).

**FIGURE 1:**
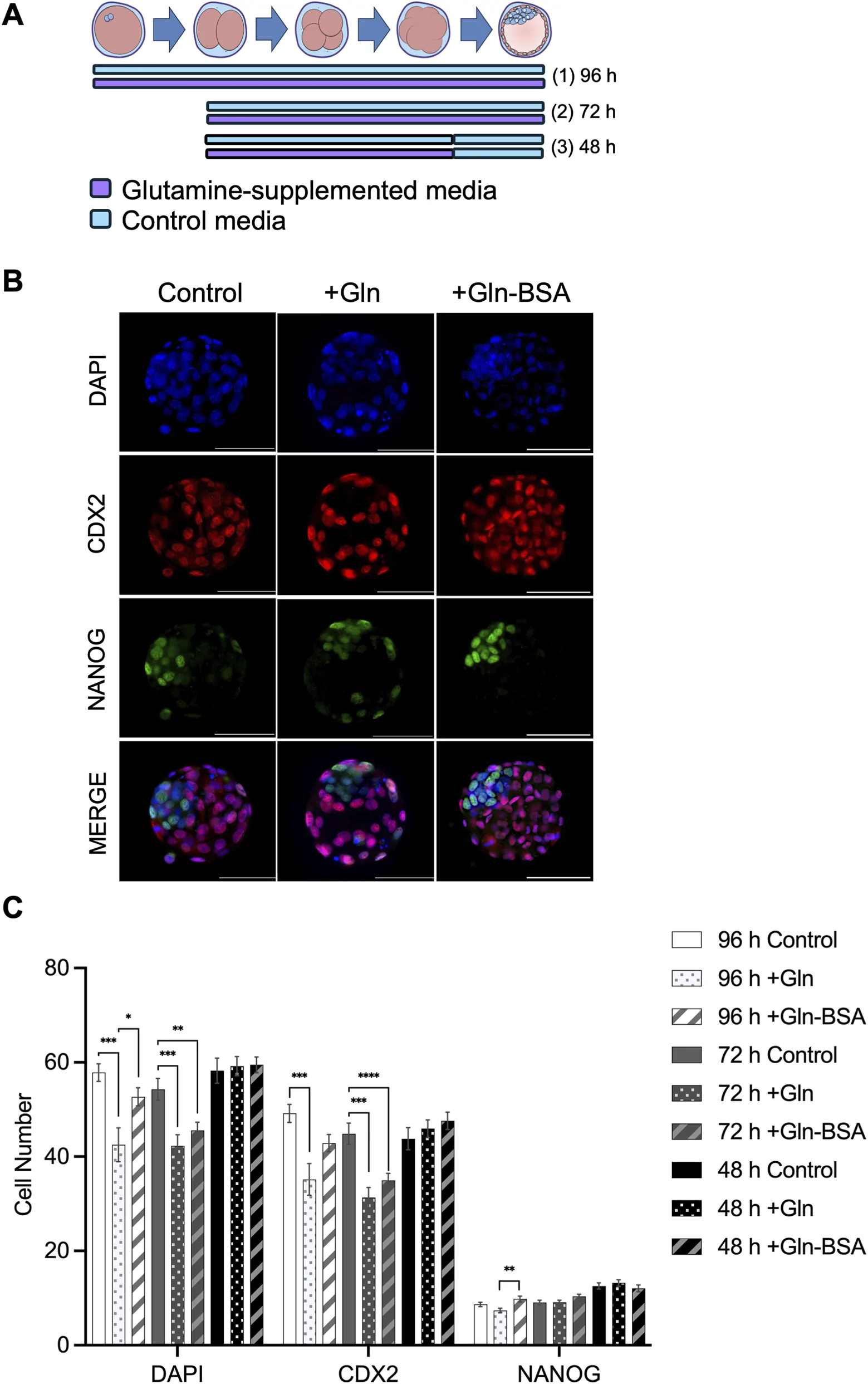
Mouse blastocyst cell distribution **(A)** Schematic overview of the experimental design in which 1-cell and 2-cell embryos were cultured in either control or glutamine-supplemented media for varying durations **(B)** Representative immunofluorescence images of CDX2, NANOG and DAPI staining in blastocysts after 72 h of culture in glutamine-supplemented media. Scale bars = 50 μm **(C)** Total cell number, CDX2-positive cells, and NANOG-positive cells in blastocysts cultured for 96 h, 72 h culture and 48 h of glutamine-supplemented media. Data are presented as mean ± SEM for each treatment group from five independent replicates. Statistical significance was assessed using one-way ANOVA followed by Tukey-Kramer’s HSD multiple comparison tests. Abbreviations: caudal type homeobox 2 (CDX2). *p < 0.05, **p < 0.01, ***p < 0.001, ****p < 0.0001.

**TABLE 1:** Mouse blastocyst development and hatching rate.

Groups	No. of embryos					Average dev. rate % ± SEM	Average hatching rate % ± SEM
	1-Cell	2-Cell	4–8 cell	Morula	Blastocyst		
96 h culture (from 1-cell until blastocyst)							
Control	53	48	48	48	47	87 ± 6.4	14 ± 3.5
+Gln	52	49	49	49	44	84 ± 5.4	4 ± 4.4
+Gln-BSA	54	51	47	47	47	84 ± 5.4	20 ± 7.6
72 h culture (from 2-cell until blastocyst)							
Control	-	100	99	99	99	100 ± 0.0	23 ± 8.9
+Gln	-	104	104	104	102	97 ± 1.7	25 ± 8.1
+Gln-BSA	-	100	99	99	99	99 ± 0.9	19 ± 4.3
48 h culture (from 2-cell until morula, changed into control media until blastocyst)							
Control	-	53	50	50	50	95 ± 1.7	16 ± 10.3
+Gln	-	53	51	51	51	96 ± 1.8	27 ± 6.3
+Gln-BSA	-	54	53	53	53	98 ± 1.9	27 ± 2.3

Development rate and hatching rate were analysed separately. Within each culture-duration experiment (96 h, 72 h and 48 h), the treatment groups were compared using one-way ANOVA. No significant differences were observed among treatment groups. (p > 0.05).

To determine the effects on cell proliferation and lineage allocation, expanded blastocysts were stained with DAPI to quantify total cell numbers. Blastocysts were immunostained with antibodies against CDX2 and NANOG to distinguish the trophectoderm (TE) and inner cell mass (ICM) as shown in Figures 1B,C.

Following 96 h of continuous culture, we compared the findings against the control embryos that developed in +Gln media had significantly reduced cell numbers compared to control (p = 0.0001). In contrast, in +Gln-BSA or the removal of BSA in increased glutamine concentration, slightly rescued the cell number between glutamine groups (+Gln vs. +Gln-BSA; p = 0.0186). To determine whether this reduction reflected changes in specific cell lineages, CDX2- and NANOG-positive cells were quantified. The decreasing number of cells was only observed in CDX2-positive or blastocyst TE cells in increased glutamine-supplemented media (+Gln; p = 0.0003) but not in NANOG-positive cells. However, comparison between the presence of BSA in glutamine-treated groups presented NANOG-positive cells were significantly different, showing a reduced value in +Gln compared to +Gln-BSA (p = 0.005). These alterations did not cause any difference shown in the distribution of the ICM:TE ratio in all groups (Supplementary Figure S1A).

A similar trend was observed after continuous embryo culture for 72 h. In both glutamine groups, a significant reduction of DAPI-positive cell numbers was seen against the controls (+Gln; p = 0.0006; +Gln-BSA p = 0.0091). Analysis of lineage allocation showed that for CDX2-positive cells, the quantity was reduced in the experimental groups compared to the control (+Gln; p = 0.0002 and +Gln-BSA; p = 0.0014). Here, the NANOG-positive cells were also not affected by the increase in glutamine concentration supplemented in culture medium (p = 0.0879) but it did show a significant increase in ICM:TE of +Gln-BSA (p = 0.0171) as shown in Supplementary Figure S1B.

Finally, we observed that the effects of culture with increased glutamine concentration were rescued after transferring back into control media at the morula stage after 48 h culture, as shown in Figure 1C. There were no changes in total cell numbers in both experimental groups compared to the control. Similar to the DAPI analysis, CDX2 and NANOG cells remained unchanged (p = 0.4468 and p = 0.4908), indicating that the reduction in blastocyst cell number induced by the alteration of glutamine concentration was reversible following culture in control medium. This was supported when the ICM:TE also exhibited no changes between groups as shown in Supplementary Figure S1C.

### 3.2 High glutamine availability increased apoptosis in mouse embryos

To determine whether reduced cell numbers were due to the cell death in embryos, we analysed the apoptosis rate of embryos at morula stage (Figure 2A). The apoptotic index was calculated using TUNEL assay, the glutamine treatment groups presented an increase in apoptotic cells with (+Gln; p = 0.0103) and without BSA (+Gln-BSA; p = 0.0092) as shown in Figures 2B,C. This implies that the elevated glutamine exposure in culture medium induces apoptosis during development.

**FIGURE 2:**
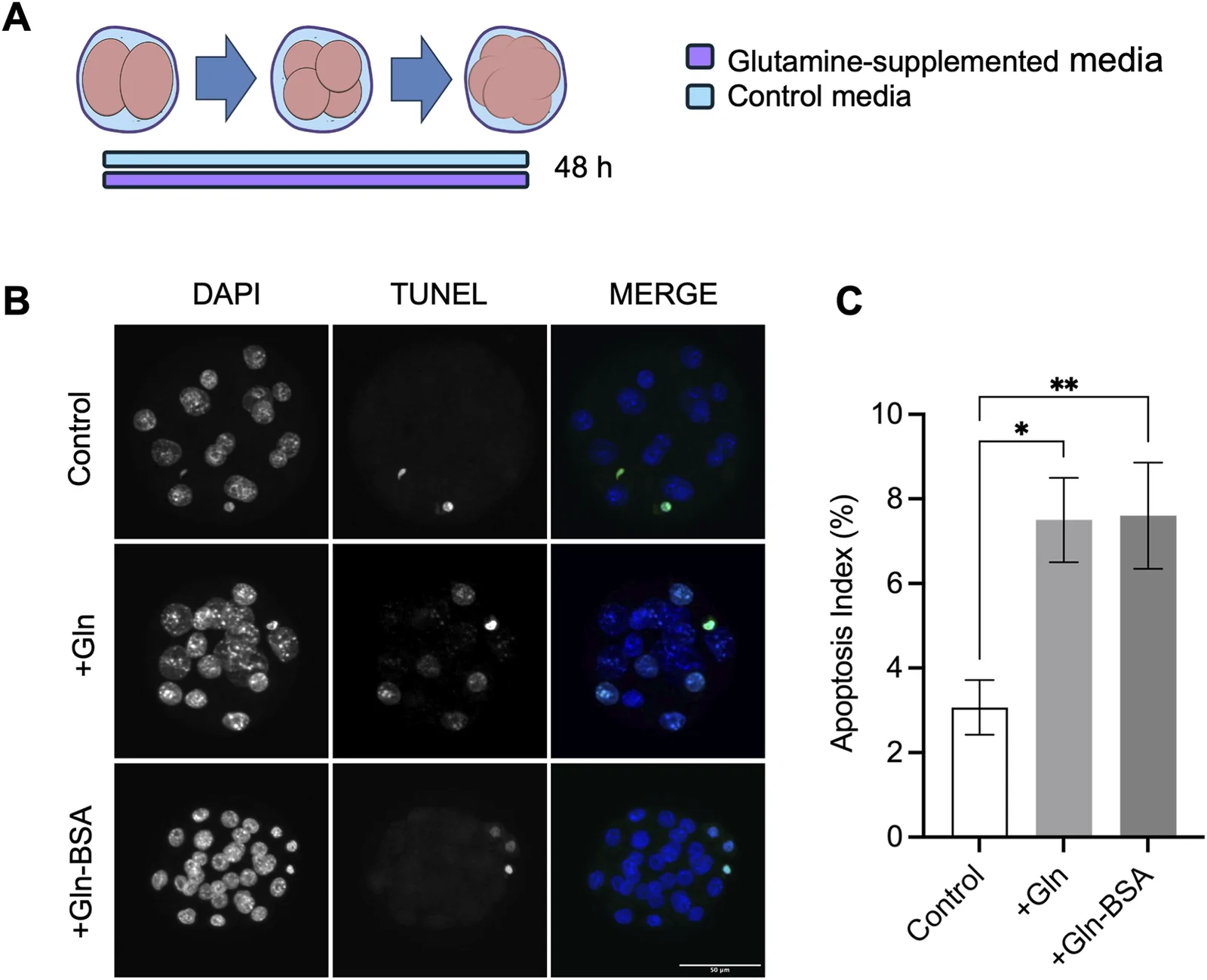
Embryo apoptosis assessment **(A)** Schematic representation of culture timeline from the 2-cell stage to the morula stage in either control or increased glutamine-supplemented media **(B)** Representative images of TUNEL assay staining in morula-stage mouse embryos following glutamine exposure. Scale bar = 50 μm **(C)** Quantification of the apoptosis index in control and glutamine-treated embryos. Data are presented as mean ± SEM of each treatment group from five independent replicates. Statistical significance was determined using one-way ANOVA followed by Tukey-Kramer’s HSD multiple comparison test. *p < 0.05, **p < 0.01.

### 3.3 Altered mitochondrial dynamics under elevated glutamine culture media

Glutamine exposure has been reported to directly influence energy metabolism through the tricarboxylic acid cycle (TCA cycle) (Devreker et al., 1998; Nesci, 2017), therefore we wanted to observe if there was any association between the increased extracellular glutamine addition and intracellular mitochondrial activity. To determine the effects of increased glutamine concentration towards the energy metabolism in embryos, we performed live-cell imaging of mitochondrial membrane potential at the morula stage (Figure 3). Embryos that developed in high glutamine availability presented lower mitochondrial membrane potential and the reduction was more severe when BSA was removed, as shown in Figure 3A (+Gln p = 0.0032; +Gln-BSA p < 0.0001).

**FIGURE 3:**
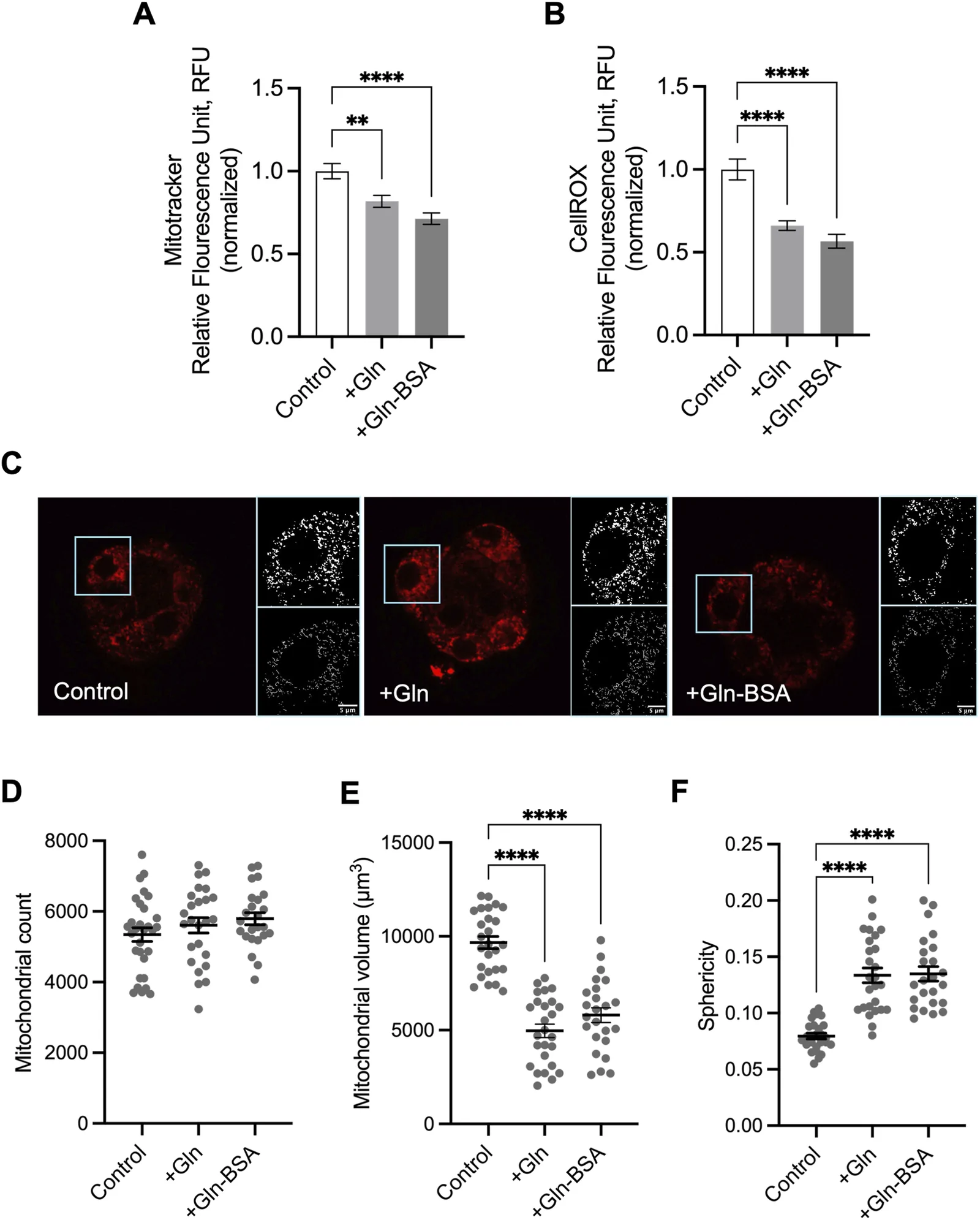
Mitochondrial metabolic and morphology assessment. Normalised relative fluorescence unit (RFU) of **(A)** mitochondrial and **(B)** CellROX™ signal intensity with glutamine supplementation **(C)** Confocal fluorescence images of mitochondrial morphology (visualised with Mitotracker™) between control and glutamine-treated culture medium. Optimised threshold images (top) and skeletonised representation of mitochondria are shown in the panels (bottom). Boxed regions are magnified. Scale bars = 5 μm. Quantification of mitochondrial network parameters compared between morula embryos cultured in control and high glutamine media: mitochondrial **(D)** count **(E)** volume (μm^3^) and **(F)** sphericity. Data are presented as mean ± SEM of each treatment group from at least three independent replicates. Statistical significance was determined using one-way ANOVA followed by Tukey-Kramer’s HSD multiple comparison test. **p < 0.01, ****p < 0.0001.

The relative intracellular ROS signals were reduced with glutamine supplementation, +Gln p < 0.0001 and +Gln-BSA p < 0.0001 (Figure 3B). Mitochondria and the reactive oxygen species (ROS) were observed together as shown in Supplementary Figure S2A, where we analysed the colocalization between Mitotracker™ and CellROX™ signals as shown in Supplementary Figure S2B. Pearson’s correlation coefficient was consistent in control and treatment groups, indicating a strong linear relationship between the mitochondrial mass and ROS signals despite culture medium alterations.

Further quantification of mitochondrial volume and morphology was conducted using confocal images to determine the effects of high glutamine in culture media, as shown in Figures 3C–F. From the representative images of the mitochondrial structures in Figure 3C, the top panel represented the optimized threshold images, and the bottom panel represented the skeletonized images used for mitochondrial analysis. Based on the skeletonized images, the mitochondrial structures from glutamine-supplemented groups (+Gln and +Gln-BSA) presented shorter single mitochondrial patterns compared to control that had longer and interconnected patterns. Mitochondrial count per embryo exhibited no significant difference between groups (Figure 3D). The morula cultured in increased glutamine-supplemented media (+Gln and +Gln-BSA) exhibited mitochondria that were smaller and more spherical compared to the control as shown in Figures 3E,F. These results suggest that glutamine supplementation induces mitochondrial fragmentation in embryonic development.

### 3.4 Perturbations in autophagy-associated process in increased glutamine availability

Glutamine has been reported to influence autophagy (Zhou et al., 2022) and consequently, autophagy influences postnatal development (Sato et al., 2025). To understand if the supplementation of increased glutamine concentration in embryo culture media affects the autophagy-associated structures, we executed two observations using DAPGreen assay and LC3-fluorescence immunostaining. DAPGreen signals, which detects both autophagosomes and autolysosomes, whereas the LC3-specific antibody detects autophagosome protein signals. We analysed the embryonic autophagy-specific structures at the same stage of development.

DAPGreen fluorescence signals were increased in +Gln-BSA (p < 0.0001) compared to control, while +Gln did not present any significant change (Figure 4A). Interestingly, there was a difference between the glutamine groups (+Gln vs. +Gln-BSA; p < 0.0001). The colocalization of mitochondria onto autophagosome and autolysosome signals presented no significant differences between treatment groups (Supplementary Figure S2D), indicating no disturbance in the mitophagy activity despite alterations in mitochondrial structures.

**FIGURE 4:**
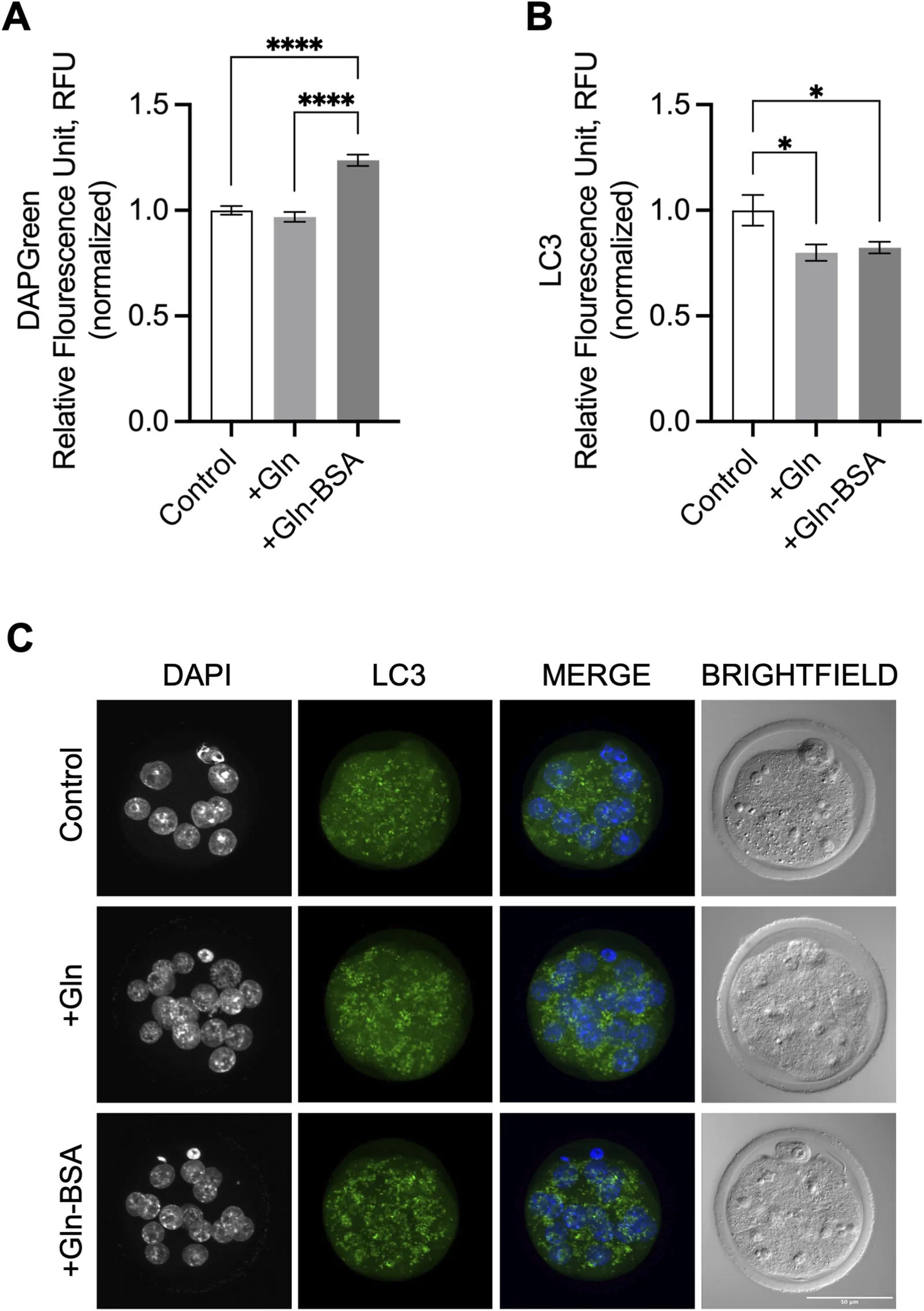
Autophagy metabolic analysis. Normalised relative fluorescence unit (RFU) of **(A)** DAPGreen and **(B)** LC3-positive autophagosomes **(C)** Representative confocal images of LC3 localisation in mouse embryos. Scale bar = 50 μm. Data are presented as mean ± SEM of each treatment group from at least three independent replicates. Statistical significance was determined using one-way ANOVA followed by Tukey-Kramer’s HSD multiple comparison test. *p < 0.05, ****p < 0.0001.

We conducted further validation by analysing a marker of LC3-positive autophagosome membranes under the same glutamine conditions at the same developmental stage as shown in Figure 4B. As depicted in Figure 4C, the fluorescence signal in glutamine treatment groups was faint compared to the control (+Gln p = 0.0124; +Gln-BSA p = 0.0301). Dot-like puncta were observed to be dispersed despite glutamine treatment in morula stage embryos. Further analysis on the LC3-specific puncta showed that the average number of LC3 puncta per embryo was also not altered, as well as the puncta volume as shown in Supplementary Figures S2E,F. Collectively, these results imply a disturbance in autophagy-related markers due to increased glutamine availability in culture medium, mostly to the autophagy-related processing and not morphologically.

### 3.5 Glutamine modulates H3K27me3 signals

To further explore whether developmental programming was accompanied by altered heterochromatin methylation patterns linked to cellular metabolism, we examined H3K27me3, a histone modification increasingly recognised to respond to mitochondrial metabolic state and implicated in metabolic disease context (Sánchez-Ceinos et al., 2024; Pladevall-Morera and Zylicz, 2022). To determine whether glutamine supplementation causes altered heterochromatin methylation patterns during embryonic development, fluorescence analyses were conducted to determine the effects of glutamine availability (Supplementary Figure S3A). Embryo heterochromatin, H3K27me3 presented a very dim signal pattern in the +Gln-BSA group (p = 0.0084); however, there was a relative reduction in the +Gln group, but not statistically significant (p = 0.4035), as shown in Supplementary Figure S3B.

### 3.6 High glutamine levels in embryo culture affect postnatal growth

In this study, we wanted to distinguish if the increased L-glutamine concentration found in αMEM contributed to the postnatal disruption found in the MEM mouse model (Ishiyama et al., 2021c). To observe the postnatal developmental effects, we produced offspring from each treatment group (Figure 5A). Birth rate upon oviductal embryo transfer showed no significant changes across groups as shown in Supplementary Table S2 (p = 0.3392). Late-gestational postnatal observation exhibited no abnormalities in offspring derived from increased glutamine culture conditions (Supplementary Figure S4A). The fetal and placental weight remained unchanged, showing no significant effects on fetal-to-placental weight as shown in Supplementary Figures S4B-D (fetal weight, p = 0.1391; placental weight, p = 0.9568 and fetal:placental, p = 0.1415).

**FIGURE 5:**
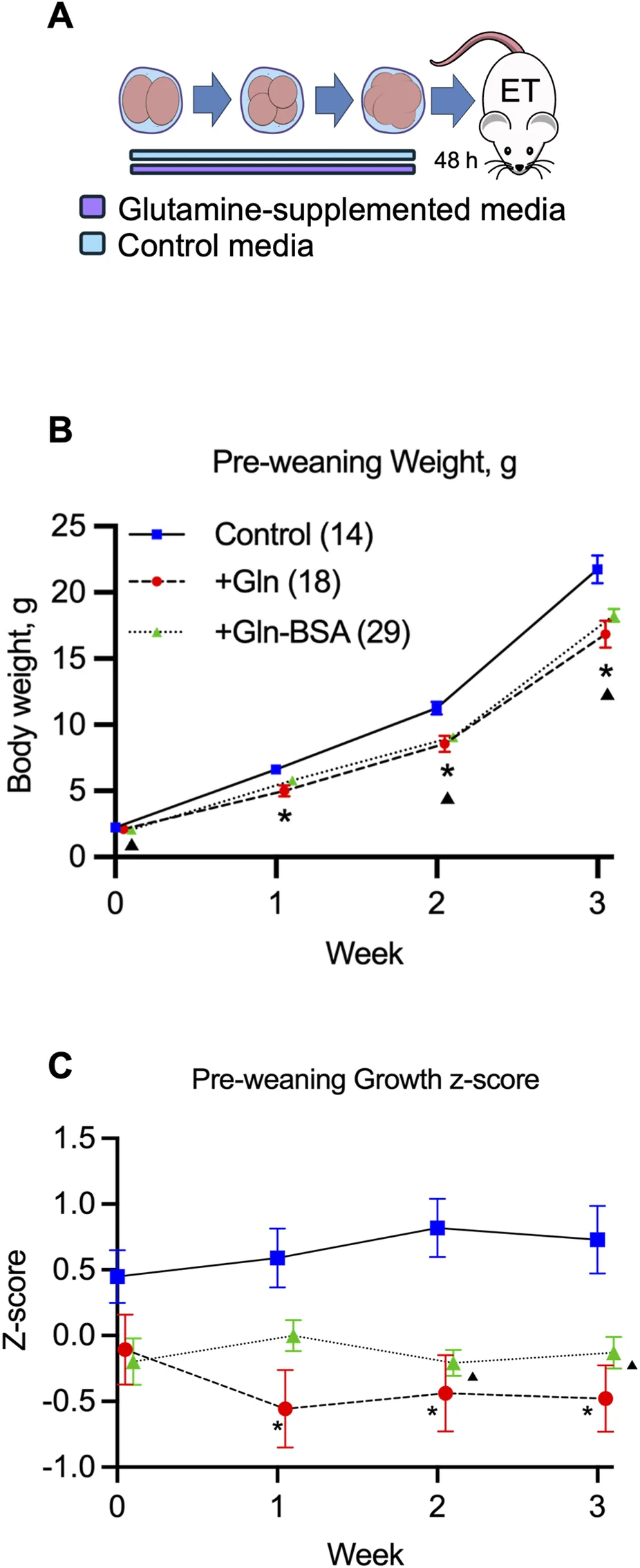
Pre-weaning offspring body weight derived from glutamine-supplemented embryo culture **(A)** Schematic representation of the embryo culture duration before transfer into pseudo-pregnant mice. Abbreviations: embryo transfer (ET) **(B)** Body weight of offspring during the pre-weaning period for each treatment group **(C)** Growth z-score analysis for pre-weaning mice. Statistical significance was determined using mixed-effects analysis followed by Tukey’s *post hoc* multiple comparison test, with significance indicated as follows: * Control vs. +Gln; ▴Control vs. +Gln-BSA. *p < 0.05*.

To further determine whether the effects of elevated glutamine levels in culture conditions persist into offspring development, offspring body weight was monitored weekly. Preliminary analysis confirmed that litter size did not significantly contribute to overall variance (p > 0.05, p = 0.850). Mean weekly weights for naturally delivered offspring without sex stratification from birth to 3-weeks old were analysed as shown in Figure 5B. Mouse offspring from glutamine-treated groups consistently had low body weight. In comparison with the control to observe the effects of increased glutamine concentration, +Gln were consistently significantly lower from week 1 until week 3 (Week 1 p = 0.0132; Week 2 p = 0.0075; Week 3 p = 0.0029). Offspring from +Gln-BSA were significantly lower than control, with a p-value of 0.0343, 0.0089 and 0.0082 on weeks 0, 2 and 3, respectively. From Week 3, body weight was observed depending on sex. After normalizing the body weights between groups, the body growth z-score before weaning presented that glutamine-treated groups (+Gln and +Gln-BSA) had lower growth z-scores than the control based on the mixed-effects analysis with *post hoc* Tukey’s test (Figure 5C). From week 1 until week 3, +Gln was significantly lower than the control (p = 0.0110, p = 0.0045 and p = 0.0056, respectively) while +Gln-BSA was significantly lower from week 2 until week 3 (p = 0.0009 and p = 0.0164).

As our study was based on previous studies establishing MEM mouse model exhibiting metabolic disorders, with these alterations identified predominantly in male offspring. To ascertain whether there is a comparable susceptibility as discovered in the MEM mouse model, the current investigation concentrated the high-fat diet on males. For male offspring, glutamine-treated groups had relatively caught up with the body weight of the control group (Figure 6A). Despite this, the Tukey’s *post hoc* test did not show any significant differences between groups, mixed-effect modelling identified significant interaction between weekly body weights and culture medium groups (p = 0.0190). Importantly, the culture media treatment correlation with weekly body weights (p = 0.0050) indicated that the postnatal growth trajectories differed among the groups, demonstrating that increased glutamine concentration during the preimplantation period influenced that longitudinal offspring growth trajectories.

**FIGURE 6:**
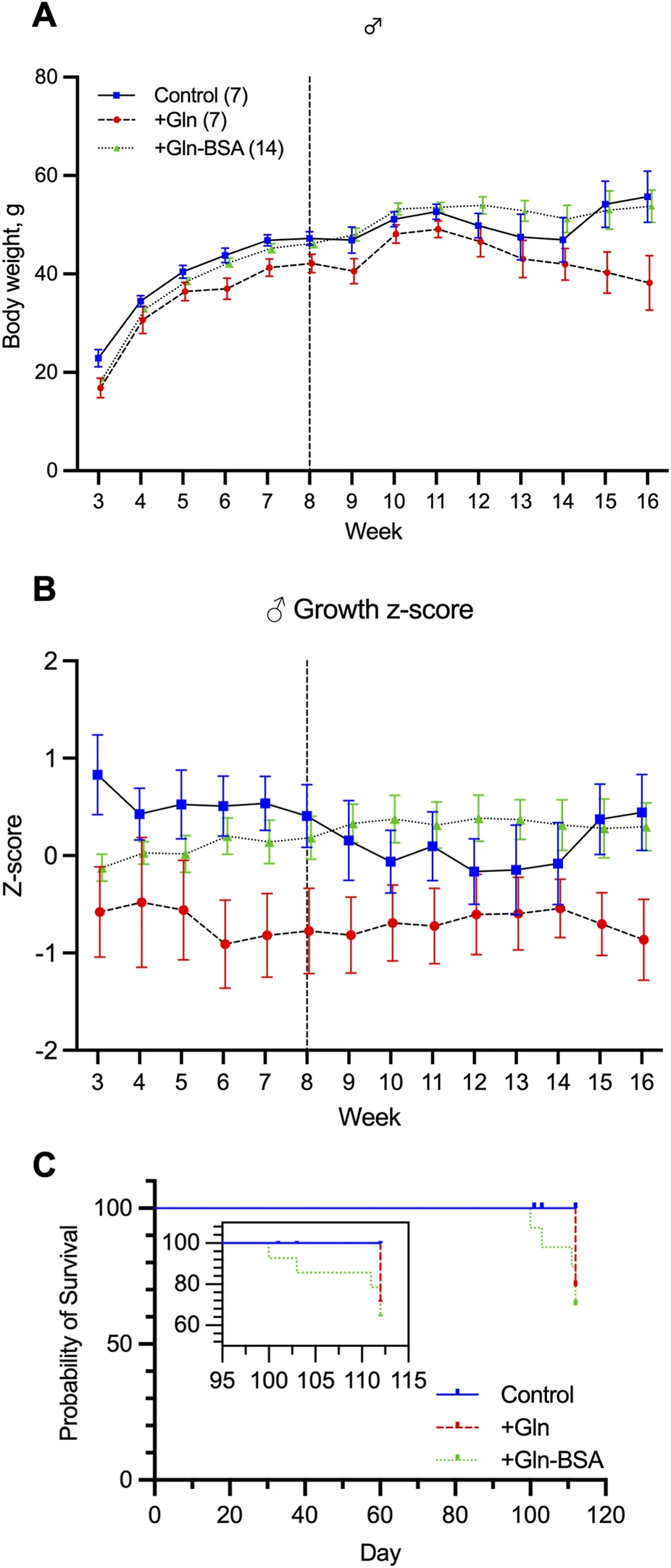
Male body weight with postnatal growth and survivability **(A)** Body weight trajectories from week 3 until week 16. The number of offspring per treatment group is indicated in the legend **(B)** Growth z-score analysis between week 3 and week 16. The dotted vertical line at week 8 indicates the initiation of high-fat diet. Statistical significance was determined using mixed-effects analysis followed by Tukey’s *post hoc* multiple comparison test (*p > 0.05*). Data are presented as mean ± SEM **(C)** Kaplan-Meier survival curves showing offspring survivability across all treatment groups.

For female offspring, a commercial diet was given throughout the experimental period. The female offspring presented a similar growth pattern, whereby offspring body weight showed significant alterations based on culture medium groups (p < 0.0001). The interaction between weekly body weights and culture medium groups were significantly altered (p < 0.0001) as well as the glutamine treatment relation with body weight trajectory (p = 0.0299) as shown in Supplementary Figure S5A. Multiple comparisons analysis presented that +Gln and +Gln-BSA initially both had a lower body weight at week 3 (+Gln, p = 0.0119; +Gln-BSA, p = 0.0246) but only +Gln continued to present lower body weight (p < 0.05). At week 14, the +Gln group was significantly lower than +Gln-BSA (p = 0.0429), suggesting a different body weight trajectory phenotype between the two glutamine treatment groups.

By normalising the body weight growth with the control group, the growth z-score for each group showed a distinct pattern depending on glutamine groups. The results showed that mouse offspring derived from +Gln group consistently had negative growth, regardless of sex (Figure 6B; Supplementary Figure S5B). This condition was more apparent in female mice, as it was found to have statistical significance in most weekly growth z-score comparisons (p < 0.05). The z-scores in male and female offspring from +Gln-BSA, presented a similar growth pattern, a slow increase after weaning as seen in Supplementary Figures S6B,S5B. Despite the absence of high-fat diet in female mice, the growth z-score showed the same trend as exhibited in male mice suggesting that the irregular body growth trajectory was not induced by the change in diet.

The overall survival was evaluated using a Kaplan-Meier survival plot based on culture media as shown in Figure 6C. The offspring of +Gln-BSA survivability to pass 100 days dropped to almost 64%, while +Gln had a 71% chance to survive. This indicates that the +Gln-BSA group showed a severe health risk.

### 3.7 Glutamine postnatal effects on glucose homeostasis

We further assessed the influence of glutamine on offspring glucose homeostasis; we performed an oral glucose tolerance test (OGTT) at week 8, 12 and 16. Male offspring fasting glucose levels (i.e. 0 min) and after 15, 30 min, 1 and 2 h of glucose loading showed all treatment groups to have slower recoveries than the control group (Figures 7A–C). At week 16, the +Gln group had a slower glucose recovery associated with a significantly higher blood glucose level at 30 min post-glucose administration (p = 0.0156). Furthermore, glucose recovery for +Gln-BSA was poorer than the control at week 12 and 16; as seen when blood glucose levels remained high even after 30 and 60 min; however, the values were not significantly different from the control group (30 min; p = 0.1598 and 60 min; p = 0.2938).

**FIGURE 7:**
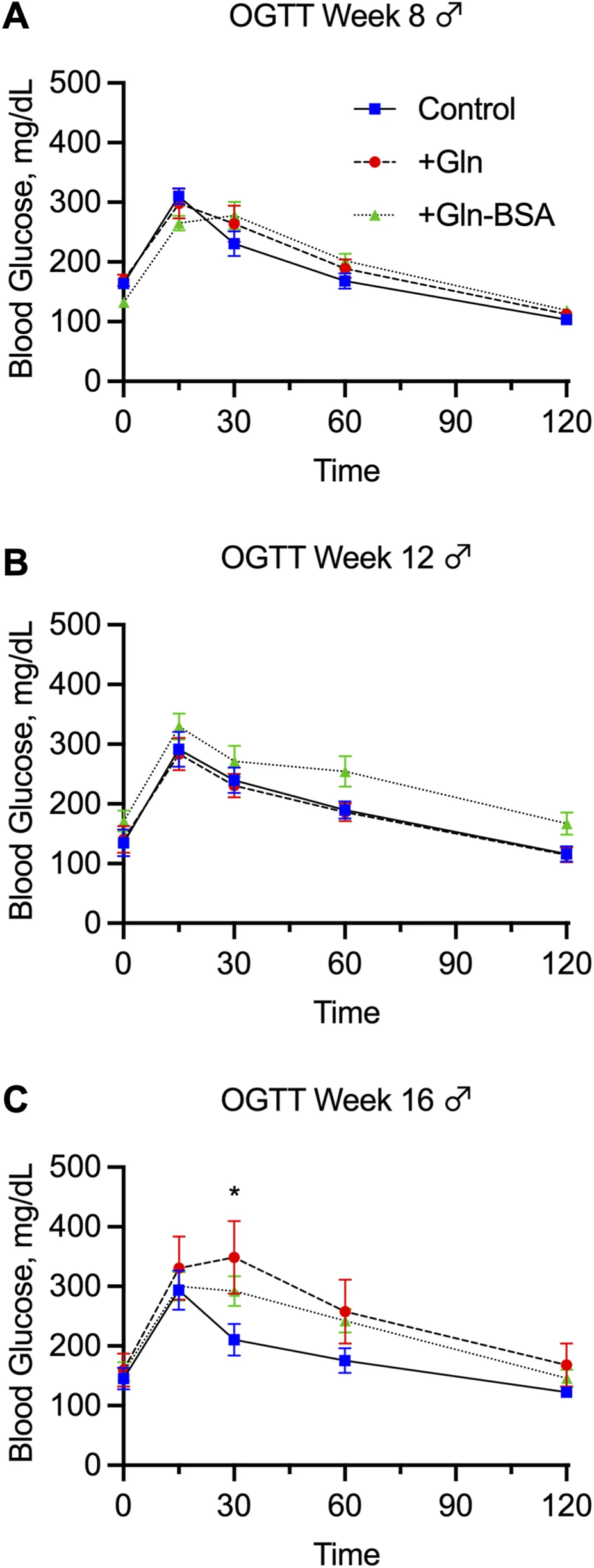
Glucose tolerance test for male mice. Blood glucose levels measured at 0, 15, 30, 60 and 120 min following oral glucose administration in male at **(A)** 8- **(B)** 12- and **(C)** 16-week of age. Data are presented as mean ± SEM. Statistical significance was determined using two-way ANOVA followed by Tukey’s *post hoc* multiple comparison test. *p < 0.05.

For female offspring, the initial glucose loading at 15 min was significantly higher in the +Gln group, but this was only seen at week 8 (Supplementary Figure S5A). As the mouse ages, the glucose tolerance did not differ between groups (Supplementary Figures S5A-C). At week 16, the glucose levels of +Gln-BSA had a slightly slower recovery than control and +Gln, as seen in the OGTT graph in Supplementary Figure S5C. At the 30 min mark, the blood glucose levels remained similar to the initial 15 min after oral glucose administration.

We also analysed the incremental area under the curve (iAUC) from the graph generated based on the oral glucose tolerance test (Supplementary Figures S6A-B). We found that the iAUC for males continuously increased as the offspring aged but reached significant differences by week 16, as shown in Supplementary Figure S6A (+Gln vs. Control; p = 0.0460). This, however, was not observed in female offspring.

## 4 Discussion

The main objective of our study was to determine the postnatal consequences of increased L-glutamine concentrations during early embryonic development. This was primarily to test the hypothesis that this component from αMEM media was causative in the subsequent postnatal phenotype of offspring from the MEM mouse model (Ishiyama et al., 2021a). The αMEM medium differs from standard embryo culture conditions in at least two relevant aspects, which are higher glutamine concentration and the absence of BSA, therefore we specifically evaluated elevated L-glutamine in the presence and absence of BSA. Our research found that elevated glutamine concentrations in culture media during early embryogenesis are associated with the reduced TE cell number, altered postnatal growth trajectories, and changes in glucose tolerance with alterations in the mitochondrial and autophagy-related dynamics. These findings support the concept that nutritional conditions during preimplantation development influence long-term developmental trajectories through adaptive early embryo metabolic reprogramming.

Our findings showed that embryos cultured in medium containing 2 mM glutamine with the presence of BSA (+Gln) exhibited persistently reduced body weight, whereas the removal of BSA (+Gln-BSA) produced a postnatal ‘catch-up’ characteristic resembling reported low protein developmental programming animal models (Nemoto et al., 2020), which supports the removal of BSA to represent reduced protein availability during embryonic development (Natsuyama et al., 1993). Maternal protein malnutrition during preimplantation has been reported to be linked with mitochondrial function (Vidyadharan et al., 2022) and metabolic diseases later in life under the DOHaD theory (Watkins et al., 2011). In the present study, the +Gln-BSA group presented lower postnatal survivability and altered glucose tolerance, which was associated with increased blastomere apoptosis, lower mitochondrial membrane potential, altered autophagosome and autolysosome markers and reduced histone methylation fluorescence pattern. Notably, a previous study using 1 mM Gln in the absence of BSA compared to 1 mM Gln with BSA presented a reduction in blastocyst cell lineage allocation associated with glucose intolerance but no significant differences in postnatal body weight (Jharna et al., 2025). Although both studies demonstrate similar effects on blastocyst development and glucose homeostasis, the absence of postnatal growth differences in the previous study suggests that the more pronounced phenotype observed here may result from the combined effect of elevated glutamine concentration and removal of BSA rather than protein depletion alone. This observed phenotype should be interpreted cautiously as the present study did not include a 1 mM glutamine without BSA control group, preventing complete separation of the individual effects of elevated glutamine from those associated with BSA depletion. Furthermore, although offspring growth was analysed without litter standardisation, preliminary analyses confirmed that litter size did not significantly contribute to overall variance. Collectively, these findings suggest that the combination of elevated glutamine concentration and reduced protein availability during embryo culture may exacerbate developmental programming associated with the metabolic diseases observed in the MEM mouse model.

The main outcomes from the elevated glutamine media were significant alterations in mitochondrial dynamics consistent with findings that glutamine availability directly influences embryonic energy metabolism (Nesci, 2017), as well as links to mitochondrial dysfunction (Bornste et al., 2023). Earlier studies have reported the perturbations of *in vitro* culture itself on mitochondrial activity (Czernik et al., 2022). Here, we extend these findings by demonstrating the alterations of culture media nutrient composition is also associated with changes in mitochondrial membrane potential and morphology. Unexpectedly, increased glutamine exposure was accompanied by reduced mitochondrial membrane potential and overall reactive oxygen species (ROS). Previous reports have described a phenomenon of “mitochondrial mild depolarisation” in embryos that limits mitochondrial ROS production while preserving developmental competence (Vyssokikh et al., 2020). Although mitochondrial depolarisation is frequently interpreted as dysfunction, reduced membrane potential may also reflect a compensatory response rather than evidence of mitochondrial damage, this can be seen by limiting excessive mitochondrial ROS generation. However, our findings did indicate that this apparent embryo adaptation was not sufficient in preserving embryo quality. Despite that most embryos were able to develop into blastocyst and eventually a viable offspring, embryos cultured under increased glutamine concentration significantly increased apoptosis and reduced total blastocyst cell number. These findings suggest that altered mitochondrial activity during preimplantation development may represent an early adaptive response to nutrient excess that supports short-term developmental survival while compromising embryo quality.

Furthermore, our findings show that in males, as early as 8-weeks of age, the glutamine-supplemented groups exhibited consistently higher blood glucose levels than the control group from 30 to 60 min after glucose loading, indicating impaired glucose regulation that becomes more apparent with age. Mitochondrial dysfunction is closely associated with diabetic symptoms (Kwak et al., 2010). The postnatal observations occurred alongside early embryonic alterations from glutamine groups with reduced mitochondrial membrane potential therefore suggests a potential association between early metabolic state. To further explore whether developmental programming was accompanied by altered heterochromatin methylation patterns linked to cellular metabolism, we examined H3K27me3, a histone modification increasingly recognised to respond to mitochondrial metabolic state and implicated in metabolic disease context (Sánchez-Ceinos et al., 2024; Pladevall-Morera and Zylicz, 2022). While we observed statistically significant reduction in H3K27me3 immunofluorescence intensity under elevated glutamine conditions accompanied by BSA removal, a similar lowered signal was also observed under elevated glutamine with BSA, which did not reach statistical significance. Therefore, the present findings cannot distinguish an independent effect of elevated glutamine from that of protein depletion or their potential interaction in regulating H3K27me3 levels. Further molecular studies measuring glutamine-derived metabolites and chromatin regulatory pathways are necessary to determine whether the epigenetic changes consistently contribute directly to later metabolic phenotypes. These findings help us conclude that elevated glutamine concentrations during preimplantation development were associated with altered mitochondrial characteristics, potentially contributing to persistent alterations in postnatal development.

In parallel with mitochondrial changes, increased glutamine concentration showed alterations in autophagy-related markers during embryonic development. Autophagy itself has a critical role in embryo development by maintaining intracellular regulation (Uechi et al., 2025) and postnatal development (Sato et al., 2025). DAPGreen signals, which detect both autophagosomes and autolysosomes, were increased in response to the addition of glutamine in culture media, whereas LC3-positive autophagosome protein signals were reduced. Additionally, there were no differences between LC3 puncta number and volume. Because these assays reflect different structures of the autophagy process and autophagic flux was not directly assessed, these findings should be interpreted cautiously and may indicate altered autophagy-related processing rather than definitive disruption of autophagic flux. Nevertheless, our observations support that excess glutamine can influence autophagy-related activity (Zhou et al., 2022) with further association of postnatal disturbances.

In our study, we found that one developmental consequence was a reduction in blastocyst trophectoderm (TE) cell numbers. Following that, we did not find any alterations in the placental or fetal weight or the fetal:placental ratio. It has been previously reported that, preimplantation embryos exhibit heightened adaptive yet aberrant TE responses vulnerable to metabolic disruptions (Alfian et al., 2025; Fleming, 2025) and may respond adaptively to nutrient imbalance. It was consistent with findings following mTOR inhibition (Ma et al., 2023) however, glutamine is generally known to induce the mTORC1 pathway rather than be an inhibitor (Lu et al., 2022). Moreover, the preimplantation supplementation of the byproduct in glutaminolysis, alpha-ketoglutarate (αKG) presented to increase TE cells (Van Nerum et al., 2025). A possible explanation is that the extracellular glutamine availability does not necessarily reflect an effective intracellular utilisation. From the mitochondrial alterations observed in the present study, included reduced mitochondrial membrane potential and fragmented mitochondrial morphology, suggests that excess glutamine in culture media may impair embryo mitochondrial function, thereby limiting efficient glutaminolysis and reducing the expected downstream anabolic signalling. Under these conditions, excess glutamine may therefore represent a state of metabolic dysregulation, resulting in a phenotype that resembles impaired mTOR activity. The contrast observations show that TE responses to increased glutamine exposures are not governed by a single pathway but instead arise from multiple interacting factors that determine the cellular activity. Further studies examining intracellular αKG abundance, glutaminolytic enzyme activity, mTORC1 activation with a detailed analysis on placental morphology and function will be required to determine whether impaired glutamine metabolism underlies the reduced TE phenotype consequently resulting in altered placental efficiency. Within the DOHaD framework, such lineage-specific responses may represent developmental trade-offs in which embryos prioritise immediate survival while establishing altered trajectories that emerge later in life.

In conclusion, our findings demonstrate that the subtle changes in in vitro culture media used in ART, such as excess glutamine, have significant impacts on embryonic development resulting in altered adult phenotypes. These results suggest that elevated glutamine exposure was linked to the alteration in the metabolic systems especially the mitochondrial and autophagy-associated dynamics, and subsequent changes in offspring growth and glucose regulation. These observations support the DOHaD concept that the preimplantation embryo responds to its nutritional environment, with consequences extending into later life. However, as L-glutamine can spontaneously degrade to ammonia as previously described (Summers et al., 2005), glutamine-derived ammonia accumulation was not directly measured during embryo culture, and its contribution to the observed phenotypes cannot be excluded. The absence of a concurrent 1 mM Gln-BSA group also limits our ability to distinguish the individual effect of elevated glutamine from BSA depletion or the potential interaction of both factors. Furthermore, the atmospheric oxygen may have contributed to elevated baseline oxidative stress even though all groups were maintained under identical *in vitro* conditions. Therefore, the present findings should be interpreted as associations between altered embryonic metabolic conditions and later phenotypes rather than direct causal pathways. Future studies integrating metabolic flux analysis, quantification of key metabolites such as α-ketoglutarate and ammonia, and functional validation of mitochondrial and autophagy pathways will be necessary to confirm the proposed molecular pathways that contribute to developmental reprogramming.

## Data Availability

The original contributions presented in the study are included in the article/Supplementary Material; further inquiries can be directed to the corresponding author.

## References

[B1] Alfian N. F. Hisamoto M. Kishigami S. (2025). Inverse embryonic responses of *in vivo* and *in vitro* fertilized mouse embryos to vitamin B supplementation during preimplantation period with limited long-term risks. Theriogenology 239, 117392. 10.1016/j.theriogenology.2025.117392 40101574

[B2] Alfian N. F. Uechi K. Morishita Y. Sato K. Yui M. Jharna J. F. (2026). How early-life programming during embryogenesis imprints cellular memory. Int. J. Mol. Sci. 27 (1), 163. 10.3390/ijms27010163 PMC12786116 41516039

[B3] Bavister B. D. (1995). Culture of preimplantation embryos: facts and artifacts. Hum. Reprod. Update 1 (2), 91–148. 10.1093/humupd/1.2.91 15726768

[B4] Bodineau C. Tomé M. Courtois S. Costa A. S. H. Sciacovelli M. Rousseau B. (2021). Two parallel pathways connect glutamine metabolism and mTORC1 activity to regulate glutamoptosis. Nat. Commun. 12 (1), 4814. 10.1038/s41467-021-25079-4 34376668 PMC8355106

[B5] Bodineau C. Tomé M. Murdoch P. D. S. Durán R. V. (2022). Glutamine, MTOR and autophagy: a multiconnection relationship. Autophagy 18 (11), 2749–2750. 10.1080/15548627.2022.2062875 35470752 PMC9629096

[B6] Bolte S. Cordelières F. P. (2006). A guided tour into subcellular colocalization analysis in light microscopy. J. Microsc. 224 (3), 213–232. 10.1111/j.1365-2818.2006.01706.x 17210054

[B7] Bornstein R. Mulholland M. T. Sedensky M. Morgan P. Johnson S. C. (2023). Glutamine metabolism in diseases associated with mitochondrial dysfunction. Mol. Cell Neurosci. 126, 103887. 10.1016/j.mcn.2023.103887 37586651 PMC10773532

[B8] Bouillon C. Léandri R. Desch L. Ernst A. Bruno C. Cerf C. (2016). Does embryo culture medium influence the health and development of children born after *in vitro* fertilization? PLOS ONE 11 (3), e0150857. 10.1371/journal.pone.0150857 27008092 PMC4805279

[B9] Brinster R. L. (1963). A method for *in vitro* cultivation of mouse ova from two-cell to blastocyst. Exp. Cell Res. 32, 205–208. 10.1016/0014-4827(63)90093-4 14094038 PMC4881842

[B10] Brinster R. L. (1971). Uptake and incorporation of amino acids by the preimplantation mouse embryo. J. Reprod. Fertil. 27 (3), 329–338. 10.1530/jrf.0.0270329 5133615

[B11] Chatot C. L. Ziomek C. A. Bavister B. D. Lewis J. L. Torres I. (1989). An improved culture medium supports development of random-bred 1-cell mouse embryos *in vitro* . J. Reproduction Fertil. 86 (2), 679–688. 10.1530/jrf.0.0860679 2760894

[B12] Chatot C. L. Lewis J. L. Torres I. Ziomek C. A. (1990). Development of 1-cell embryos from different strains of mice in CZB medium. Biol. Reprod. 42 (3), 432–440. 10.1095/biolreprod42.3.432 2111184

[B13] Chaudhry A. Shi R. Luciani D. S. (2020). A pipeline for multidimensional confocal analysis of mitochondrial morphology, function, and dynamics in pancreatic β-cells. Am. J. Physiol. Endocrinol. Metab. 318 (2), E87–e101. 10.1152/ajpendo.00457.2019 31846372 PMC7052579

[B14] Chen P. R. Redel B. K. Spate L. D. Ji T. Salazar S. R. Prather R. S. (2018). Glutamine supplementation enhances development of in vitro-produced porcine embryos and increases leucine consumption from the medium. Biol. Reprod. 99 (5), 938–948. 10.1093/biolre/ioy129 29860318 PMC6297286

[B15] Czernik M. Winiarczyk D. Sampino S. Gręda P. Parillo S. Modliński J. A. (2022). Mitochondrial function and intracellular distribution is severely affected in *in vitro* cultured mouse embryos. Sci. Rep. 12 (1), 16152. 10.1038/s41598-022-20374-6 36167966 PMC9515144

[B16] Devreker F. Winston R. M. Hardy K. (1998). Glutamine improves human preimplantation development *in vitro* . Fertil. Steril. 69 (2), 293–299. 10.1016/s0015-0282(97)00463-9 9496344

[B17] Eckert J. J. Porter R. Watkins A. J. Burt E. Brooks S. Leese H. J. (2012). Metabolic induction and early responses of mouse blastocyst developmental programming following maternal low protein diet affecting life-long health. PLoS One 7 (12), e52791. 10.1371/journal.pone.0052791 23300778 PMC3531326

[B18] Fleming T. P. (2025). Preimplantation trophectoderm: a 'quick-fix' protector for embryo survival? Dev. Biol. 517, 278–285. 10.1016/j.ydbio.2024.10.006 39481626

[B19] He Y. Hakvoort T. B. Vermeulen J. L. Lamers W. H. Van Roon M. A. (2007). Glutamine synthetase is essential in early mouse embryogenesis. Dev. Dyn. 236 (7), 1865–1875. 10.1002/dvdy.21185 17557305

[B20] Holecek M. (2013). Side effects of long-term glutamine supplementation. JPEN J. Parenter. Enter. Nutr. 37 (5), 607–616. 10.1177/0148607112460682 22990615

[B21] Ishiyama S. Kimura M. Nakagawa T. Fujimoto Y. Uchimura K. Kishigami S. (2021a). Development of the diabetic kidney disease mouse model culturing embryos in alpha-Minimum essential medium *in vitro,* and feeding barley diet attenuated the pathology. Front. Endocrinol. (Lausanne) 12, 746838. 10.3389/fendo.2021.746838 34867790 PMC8634848

[B22] Ishiyama S. Kimura M. Umihira N. Matsumoto S. Takahashi A. Nakagawa T. (2021b). Consumption of barley ameliorates the diabetic steatohepatitis and reduces the high transforming growth factor beta expression in mice grown in alpha-minimum essential medium *in vitro* as embryos. Biochem. Biophys. Rep. 27, 101029. 10.1016/j.bbrep.2021.101029 34136664 PMC8181192

[B23] Ishiyama S. Kimura M. Umihira N. Matsumoto S. Takahashi A. Nakagawa T. (2021c). Mice derived from *in vitro* αMEM-cultured preimplantation embryos exhibit postprandial hyperglycemia and higher inflammatory gene expression in peripheral leukocytes. Biosci. Biotechnol. Biochem. 85 (5), 1215–1226. 10.1093/bbb/zbab023 33587104

[B24] Jharna J. F. Bari M. W. Alfian N. F. Kishigami S. (2025). The absence of bovine serum albumin (BSA) in preimplantation culture media impairs embryonic development and induces metabolic alterations in mouse offspring. Int. J. Mol. Sci. 26 (14), 6989. 10.3390/ijms26146989 PMC12295788 40725236

[B25] Kleijkers S. H. van Montfoortvan A. P. Bekers O. Coonen E. Derhaag J. G. Evers J. L. (2016). Ammonium accumulation in commercially available embryo culture media and protein supplements during storage at 2-8°C and during incubation at 37°C. Human reproduction (Oxford, England) 31 (6), 1192–1199. 10.1093/humrep/dew059 27052500

[B26] Kwak S. H. Park K. S. Lee K. U. Lee H. K. (2010). Mitochondrial metabolism and diabetes. J. Diabetes Investig. 1 (5), 161–169. 10.1111/j.2040-1124.2010.00047.x 24843427 PMC4020716

[B27] Lane M. Gardner D. K. (1994). Increase in postimplantation development of cultured mouse embryos by amino acids and induction of fetal retardation and exencephaly by ammonium ions. J. Reprod. Fertil. 102 (2), 305–312. 10.1530/jrf.0.1020305 7861382

[B28] Lane M. Hooper K. Gardner D. K. (2001). Effect of essential amino acids on mouse embryo viability and ammonium production. J. Assist. Reprod. Genet. 18 (9), 519–525. 10.1023/a:1016657228171 11665668 PMC3455733

[B29] Lu V. Roy I. J. Torres A. Jr. Joly J. H. Ahsan F. M. Graham N. A. (2022). Glutamine-dependent signaling controls pluripotent stem cell fate. Dev. Cell 57 (5), 610–623. 10.1016/j.devcel.2022.02.003 35216682 PMC8930616

[B30] Ma C. Li Q. Yang Y. Ge L. Cai J. Wang J. (2023). mTOR hypoactivity leads to trophectoderm cell failure by enhancing lysosomal activation and disrupting the cytoskeleton in preimplantation embryo. Cell and Biosci. 13 (1), 219. 10.1186/s13578-023-01176-3 PMC10688112 38037142

[B31] Natsuyama S. Noda Y. Narimoto K. Mori T. (1993). Role of protein supplements in the culture of mouse embryos. Theriogenology 40 (1), 149–157. 10.1016/0093-691x(93)90348-9 16727301

[B32] Nemoto T. Nakakura T. Kakinuma Y. (2020). Elevated blood pressure in high-fat diet-exposed low birthweight rat offspring is Most likely caused by elevated glucocorticoid levels due to abnormal pituitary negative feedback. PLOS ONE. 15 (8), e0238223. 10.1371/journal.pone.0238223 32853260 PMC7451543

[B33] Nesci S. (2017). Glucose and glutamine in the mitochondrial oxidative metabolism of stem cells. Mitochondrion 35, 11–12. 10.1016/j.mito.2017.04.004 28438612

[B34] Peral-Sanchez I. Hojeij B. Ojeda D. A. Steegers-Theunissen R. P. M. Willaime-Morawek S. (2021). Epigenetics in the uterine environment: how maternal diet and ART may influence the epigenome in the offspring with long-term health consequences. Genes (Basel) 13 (1), 31. 10.3390/genes13010031 PMC8774448 35052371

[B35] Pladevall-Morera D. Zylicz J. J. (2022). Chromatin as a sensor of metabolic changes during early development. Front. Cell Dev. Biol. 10, 10–2022. 10.3389/fcell.2022.1014498 36299478 PMC9588933

[B36] Sánchez-Ceinos J. Hussain S. Khan A. W. Zhang L. Almahmeed W. Pernow J. (2024). Repressive H3K27me3 drives hyperglycemia-induced oxidative and inflammatory transcriptional programs in human endothelium. Cardiovasc Diabetol. 23 (1), 122. 10.1186/s12933-024-02196-0 PMC10998410 38580969

[B37] Sato K. Koide I. Bari M. Kishigami S. (2025). Chloroquine mitigates long-term effects of *in vitro* culture in mouse embryos. Front. Cell Dev. Biol. 13, 1640986. 10.3389/fcell.2025.1640986 40874021 PMC12378626

[B38] Sinawat S. Hsaio W. C. Flockhart J. H. Kaufman M. H. Keith J. West J. D. (2003). Fetal abnormalities produced after preimplantation exposure of mouse embryos to ammonium chloride. Hum. Reprod. 18 (10), 2157–2165. 10.1093/humrep/deg429 14507838

[B39] Summers M. C. McGinnis L. K. Lawitts J. A. Biggers J. D. (2005). Mouse embryo development following IVF in media containing either L-glutamine or glycyl-L-glutamine. Hum. Reprod. 20 (5), 1364–1371. 10.1093/humrep/deh756 15705624

[B40] Tatebe T. Pham D. Q. Ogura A. Inoue K. (2025). mTORC1-dependent suppression of autophagic activity in somatic cell nuclear transfer mouse embryos. Reproduction (Cambridge, England) 170 (6), e250338. 10.1530/rep-25-0338 41150827 PMC12910570

[B41] Uechi K. Koide I. Kanie S. Yamazaki T. Kishigami S. (2025). Regulation of autophagy and its role in late preimplantation during mouse embryo development. Sci. Rep. 15 (1), 26163. 10.1038/s41598-025-11359-2 PMC12274435 40681589

[B42] Van Nerum K. Wenzel A. Argemi-Muntadas L. Kafkia E. Drews A. Brun I. S. (2025). α-Ketoglutarate promotes trophectoderm induction and maturation from naive human embryonic stem cells. Nat. Cell Biol. 27 (5), 749–761. 10.1038/s41556-025-01658-1 40269259 PMC12081308

[B43] Velazquez M. A. Idriss A. Chavatte-Palmer P. Fleming T. P. (2023). The Mammalian preimplantation embryo: its role in the environmental programming of postnatal health and performance. Anim. Reprod. Sci. 256, 107321. 10.1016/j.anireprosci.2023.107321 37647800

[B44] Vidyadharan V. A. Betancourt A. Smith C. Yallampalli C. Blesson C. S. (2022). Prenatal low-protein diet affects mitochondrial structure and function in the skeletal muscle of adult female offspring. Nutrients 14 (6), 1158. 10.3390/nu14061158 35334815 PMC8954615

[B45] Villar V. H. Durán R. V. (2017). Glutamoptosis: a new cell death mechanism inhibited by autophagy during nutritional imbalance. Autophagy 13 (6), 1078–1079. 10.1080/15548627.2017.1299315 28296535 PMC5486366

[B46] Vyssokikh M. Y. Holtze S. Averina O. A. Lyamzaev K. G. Panteleeva A. A. Marey M. V. (2020). Mild depolarization of the inner mitochondrial membrane is a crucial component of an anti-aging program. Proc. Natl. Acad. Sci. 117 (12), 6491–6501. 10.1073/pnas.1916414117 32152094 PMC7104298

[B47] Watkins A. J. Lucas E. S. Wilkins A. Cagampang F. R. A. Fleming T. P. (2011). Maternal periconceptional and gestational low protein diet affects mouse offspring growth, cardiovascular and adipose phenotype at 1 year of age. PLOS ONE 6 (12), e28745. 10.1371/journal.pone.0028745 22194901 PMC3240629

[B48] Zhao M.-H. Kim N.-H. Cui X.-S. (2016). GlutaMAX prolongs the shelf life of the culture medium for porcine parthenotes. Theriogenology 85 (3), 368–375. 10.1016/j.theriogenology.2015.08.014 26462658

[B49] Zhou J. Chen H. Du J. Tai H. Han X. Huang N. (2022). Glutamine availability regulates the development of aging mediated by mTOR signaling and autophagy. Front. Pharmacol. 13, 924081. 10.3389/fphar.2022.924081 35860029 PMC9289448

[B50] Zuo Z. Niu Z. Liu Z. Ma J. Qu P. Qiao F. (2020). The effects of glycine-glutamine dipeptide replaced l-glutamine on bovine parthenogenetic and IVF embryo development. Theriogenology 141, 82–90. 10.1016/j.theriogenology.2019.09.005 31518732

