# Supplementary material for: Excess glutamine in embryo culture media alters mitochondrial and autophagy-related dynamics associated with impaired postnatal development
Source: Front Cell Dev Biol. 2026 Sep 17;14:1920196. doi: 10.3389/fcell.2026.1920196 (PMC13627324; doi:10.3389/fcell.2026.1920196)
Supplement: Supplementary file 1 [file Table1.docx]

Supplementary Material

# 1 Table S1. Culture media compositions (mg/200mL)

| Reagent | mCZB | +Gln | +Gln-BSA |
| --- | --- | --- | --- |
| NaCl | 952.00 | 952.00 | 952.00 |
| KCl | 72.00 | 72.00 | 72.00 |
| MgSO₄・7H₂O | 58.00 | 58.00 | 58.00 |
| KH₂PO₄ | 32.00 | 32.00 | 32.00 |
| EDTA・2NA | 8.00 | 8.00 | 8.00 |
| NaHCO₃ | 422.00 | 422.00 | 422.00 |
| Na-lactate | 1.06 | 1.06 | 1.06 |
| **L-Glutamine** | **30.00** | **60.00** | **60.00** |
| D-Glucose | 200.00 | 200.00 | 200.00 |
| Na-pyruvate | 6.00 | 6.00 | 6.00 |
| PenicillinG | 10.00 | 10.00 | 10.00 |
| Streptomycin | 14.00 | 14.00 | 14.00 |
| CaCl₂・2H₂O | 50.00 | 50.00 | 50.00 |
| BSA | 1000.00 | 1000.00 | - |
| Phenol red | ~0.1 | ~0.1 | ~0.1 |

# 2 Table S2. Oviductal transfer for offspring production of the treatment groups

| Groups | No. of transferred embryos | No. female recipients | No. live offspring | Average birth rate, % ± SEM | Average birth weight, g ± SEM |
| --- | --- | --- | --- | --- | --- |
| Control | 85 | 9 | 14 | 16 ± 9.74 | 2.24 ± 0.04 |
| +Gln | 68 | 8 | 18 | 26 ± 15.65 | 2.12 ± 0.06 |
| +Gln-BSA | 85 | 9 | 29 | 37 ± 12.36 | 2.09 ± 0.04 |
| Birth rate and birth weight were analysed separately using one-way ANOVA to compare between the treatment groups. No significant differences were observed for either birth rate or birth weight. (*p > 0.05)*. | | | | | |

# 3 Table S3. High-fat diet composition (g/kg)

| Content | Western Diet (F2WTD) |
| --- | --- |
| Casein | 198.2 |
| L-cysteine | 3 |
| β-corn starch | 37.458 |
| α-corn starch | 12.5 |
| Sucrose | 340 |
| Maltodextrin | 99.8 |
| Soybean oil | 10 |
| Unsalted butter | 200 |
| Cholesterol | 1.5 |
| Cellulose powder | 50 |
| Mineral mixture, AIN93G | 35 |
| Vitamin mixture, AIN93G | 10 |
| Choline bitartrate | 2.5 |
| t-Butylhydroquinone | 0.042 |
| Total (g) | 1000 |
